# GPs’ views of prescribing beta- blockers for people with anxiety disorders: a qualitative study

**DOI:** 10.3399/BJGP.2024.0091

**Published:** 2024-09-17

**Authors:** Charlotte Archer, David Kessler, Nicola Wiles, Carolyn A Chew-Graham, Katrina Turner

**Affiliations:** Centre for Academic Mental Health, University of Bristol, Bristol Medical School, Bristol.; Centre for Academic Mental Health, University of Bristol, Bristol Medical School, Bristol.; Centre for Academic Mental Health, University of Bristol, Bristol Medical School, Bristol.; School of Medicine, Keele University, Keele.; Centre for Academic Primary Care, University of Bristol, Bristol Medical School; National Institute for Health and Care Research Applied Research Collaboration West, University Hospitals Bristol NHS Foundation Trust, Bristol.

**Keywords:** anxiety disorders, beta-blockers, general practice, prescribing, primary health care, qualitative research

## Abstract

**Background:**

Between 2003 and 2018, incident prescriptions of beta-blockers for anxiety increased substantially, particularly for young adults. National Institute for Health and Care Excellence guidance for anxiety does not recommend beta-blockers, probably due to a lack of evidence to support such use. Recent reports have highlighted the potential risks of beta-blockers.

**Aim:**

To understand when and why GPs prescribe beta-blockers for people with anxiety.

**Design and setting:**

In-depth interviews with 17 GPs in Bristol and the surrounding areas.

**Method:**

Interviews were held by telephone or video call. A topic guide was used to ensure consistency across interviews. Interviews were audio-recorded, transcribed verbatim, and analysed thematically.

**Results:**

Many GPs viewed beta-blockers as ‘low risk’, particularly for young adults. Some GPs viewed beta-blockers as an alternative to benzodiazepines, acting quickly and not leading to dependence. GPs reflected that some patients appeared to want an ‘immediate fix’ to their symptoms, which GPs thought beta-blockers could potentially offer. This is salient in light of substantial waiting lists for talking therapies and delays in antidepressants taking effect. GPs described how some patients seemed more willing to try beta-blockers than antidepressants, as patients did not perceive them as ‘mental health drugs’ and therefore viewed them as potentially more acceptable and less stigmatising. Further, GPs viewed beta-blockers as ‘patient-led’, with patients managing their own dose and frequency, without GP input.

**Conclusion:**

Many GPs believe that beta-blockers have a role to play in the management of anxiety. Given recent increases in the prescribing of these drugs in primary care, there is a need to assess their safety and effectiveness as a treatment for people with anxiety disorders.

## Introduction

Anxiety is a common mental health problem (UK prevalence is 7.2%)^1^ and is usually managed in primary care.^2^ Selective serotonin reuptake inhibitors (SSRIs), a type of antidepressant, are the standard drug prescribed by GPs to treat anxiety, yet only around 50% of patients respond to them.^3^ Alternative pharmacological treatments include benzodiazepines or pregabalin, but there are concerns around dependency.^4^^,^^5^ The beta-blocker propranolol is licensed for the management of anxiety,^6^ and incident primary care propranolol prescriptions for patients with anxiety, particularly for young adults aged 18–35 years, increased substantially between 2003 and 2018 (for all adults aged ≥18 years there was an increase from 2.3/1000 to 4.1/1000 person–years-at-risk).^7^ This is in line with the overall increase in prescribing for anxiety and depression, and may reflect an increasing sense in both GPs and the general population of the need for treatment of these disorders.^8^ However, beta-blockers do not feature in the National Institute for Health and Care Excellence (NICE) anxiety guidelines,^9^ and there is no clinical guidance detailing when and how they should be used for anxiety. This may be due to the inconclusive evidence about the benefit of beta-blockers in the treatment of anxiety, with reviews concluding that more high-quality evidence is needed.^10^^,^^11^

Further, a UK Health Services Safety Investigations Body report published in 2020^12^ highlighted a potential unacknowledged risk of toxicity in overdose with propranolol, and risks when taken alongside antidepressants. The report recommended that British National Formulary (BNF) and NICE guidance were reviewed and updated. In 2022, propranolol was added to the Advisory Council on the Misuse of Drugs’ watch list, which lists drugs considered to be increasingly widely available for misuse through diversion or illicit supply.^13^ The National Poisons Information Service annual report^14^ states that, in 2022/2023, 321 patients intentionally overdosed on propranolol, of whom one-third (*n* = 108) were prescribed the drug for anxiety. In the same year, 539 patients overdosed on antidepressants and 509 on benzodiazepines (prescribed for any indication).^15^^,^^16^

**Table:** How this fits in

Beta-blockers are licensed for managing the symptoms of anxiety, and new prescriptions for patients with anxiety have increased substantially in recent years. However, National Institute for Health and Care Excellence guidance for anxiety does not recommend beta-blockers as a treatment for anxiety, and recent reports have highlighted risks associated with the beta-blocker propranolol. Our research found that GPs prescribe beta-blockers for anxiety because they consider them to be low risk, a quicker solution than other treatments, and useful for managing associated physical symptoms.

There has been some qualitative research into the use of propranolol in medical and nursing students for test anxiety.^17^^,^^18^ However, there are a lack of qualitative data reporting primary care practitioner or patient views on the use of beta-blockers for people with anxiety. With increased prescribing of beta-blockers in primary care and potential unrecognised risk, there is a need to understand when and why GPs are prescribing these drugs for anxiety. This study therefore explored GPs’ views of prescribing beta-blockers for people with anxiety.

## Method

### Recruitment and sampling

GPs were recruited through general practices in Bristol and the surrounding area. Practices were informed about the study by the local Clinical Research Network (CRN) and the Bristol, North Somerset and South Gloucestershire Integrated Care Board (ICB). An ‘infographic’ invitation was emailed to all research active practices in Bristol, North Somerset and South Gloucestershire, which were then passed on to GPs working in their service. GPs who were interested in taking part in the study contacted the research team directly by email or telephone, and were then sent a study invitation and information sheet. GPs who were willing to be interviewed after reading the information sheet were contacted to arrange an interview time and date. Participating GPs were reimbursed with £40 to thank them for their time.

As data collection progressed, the research team liaised with GP practices via the CRN and ICB networks to promote the study to groups of GPs who were underrepresented, according to the sociodemographics of the GPs who had already been interviewed and the practices they worked in, in terms of the practice deprivation decile and the sociodemographic characteristics of their patients.

### Data collection

Semi-structured interviews were carried out by telephone or video call by the lead author, who is a health service researcher experienced in interviewing practitioners. She explained her background to participants before the interview and recorded reflexive notes throughout the process. A topic guide (see Supplementary Box S1 for details) was used to ensure consistency across interviews. It was based on the aims of the study and informed by relevant literature and discussions with research team members and the study’s patient and public involvement (PPI) group. It included questions about what types of anxiety GPs might prescribe beta-blockers for, and their views on the frequency, duration, and risks of prescribing. After each interview, GPs were asked to complete a brief demographic questionnaire, the information from which was then used to describe those interviewed and considered during analysis of the data.

### Data analysis

Data collection and analysis proceeded in parallel so that data collection would end when no new themes were identified in the later interviews and there were sufficient data to significantly contribute to understanding. It also allowed insights from early interviews to inform later data collection. For this reason, the guide was slightly revised as data collection progressed.

All the interviews were audio-recorded using an encrypted voice recorder, transcribed professionally, fully anonymised, and then checked for accuracy. Following the steps defined by Braun and Clarke,^19^ data were analysed with reflexive thematic analysis. After four interviews had taken place, two investigators read and re-read the transcripts from these interviews to identify possible codes and then met to compare and discuss their coding and interpretation of the data. In discussion with clinical members of the research team, a preliminary coding framework was developed, and this was revised as new codes were identified in subsequent transcripts. This framework was then independently applied by the two researchers to another subset of transcripts from later interviews, with new codes added as needed. The two researchers then met again to discuss their coding and interpretation of the data, with a further revision of the coding framework. Transcripts that had previously been coded were re-coded by the lead author where necessary. Throughout the analysis process, data and the interpretation of the data were discussed within the team.

Once all transcripts had been read, re-read, and coded (and re-coded where necessary), they were electronically coded in NVivo (version 12), so that data relating to each code could be easily visualised and then used to produce a code report. Data in the code report extracted were then read and re-read to identify key themes. Using an approach based on framework analysis, interview extract data were summarised in a table where each row represented individual interviewees and each column represented specific codes.^20^ This table was used to identify patterns between and within interviews, and to identify deviant cases. Themes were visualised using a mind-map created by the lead author, and discussed with the wider team, which included clinicians, alongside interview extract data. Themes were revised and refined as a result of this discussion. The reflexive notes were considered in the thematic approach, including the influence of the researchers’ own professional backgrounds, research interests, and career stages on data analysis. Where deviant cases were identified in the main themes, they are reported as the views of ‘a few GPs’ rather than ‘many’ or ‘most’.

### Patient and public involvement

Four PPI contributors with lived experience of anxiety attended a meeting to comment on initial ideas for the study. Two of these individuals contributed to the content of the interview topic guides, and questions asking practitioners how they explain beta-blockers to patients were included as a result. Twenty months later, four individuals, including the two who had helped to develop the topic guide, met to comment on the study findings. They felt that the results were important and relevant, and agreed with the researchers’ interpretation of the data.

## Results

Seventeen GPs from 10 practices were interviewed between August 2022 and February 2023 (Table 1). Around two-thirds of the GPs interviewed were female (*n* = 11, 65%), and the mean age was 46.7 years (standard deviation 8.9). Those interviewed had been consulting in general practice between 6 months and 30 years, and one GP reported having an additional qualification in mental health. The interviews lasted between 20 and 34 minutes.

**Table 1.** Characteristics of GPs interviewed and practice deprivation deciles

**Characteristic**		***n***
**Sex**	Female	11
	Male	6

**Age, years**	30–39	6
	40–49	4
	50–59	6
	≥60	1

**Ethnicity (self-disclosed)**	White	14
	Asian	2
	Mixed	1

**Role in practice**	Partner	8
	Salaried GP	9

**Practice deprivation score^a^**	1–3	5
	4–7	4
	8–10	8

a *Deprivation score for the practice patient population where 1 indicates the most deprived patient population and 10 the least deprived. Taken from the National General Practice Profiles website,^21^ which calculated scores based on the* English indices of deprivation 2015.*22*

Findings from the interviews are presented in three main areas (pragmatic prescribing, safety-driven prescribing, and patient-driven prescribing), with seven subheadings (Box 1). These subheadings reflect themes identified in the data, that were factors in GPs prescribing beta-blockers for patients with anxiety.

**Box 1.** Themes identified in the interviews relating to GP prescribing of beta-blockers

**Pragmatic prescribing** Beta-blockers are an option for managing symptoms of anxiety Beta-blockers are licensed for use in anxiety **Safety-driven prescribing** Beta-blockers are ‘low risk’ Beta-blockers as an alternative to benzodiazepines **Patient-driven prescribing** Beta-blockers have an ‘immediate’ effect Beta-blockers are not ‘mood-altering’ Beta-blockers are a ‘patient-managed’ treatment

### Pragmatic prescribing

#### Beta-blockers are an option for managing symptoms of anxiety

GPs explained there were two types of anxiety for which they might prescribe a beta-blocker: situational anxiety and generalised anxiety disorder with physical symptoms. However, some GPs reflected these were on a continuum, whereby patients often had symptoms of anxiety that were related to certain situations, such as social interactions or related to work, that they might experience on a daily basis.

GPs noted that beta-blockers were particularly useful for situational anxiety, such as during exams or performances, or for anxiety related to social situations, including those with generalised anxiety disorder that might be exacerbated by certain situations:

*‘It is very good for situational* [anxiety]*, which can include some social anxiety … meeting up with friends when they first start university or whatever … and generalised anxiety, people have their ups and their downs, and there are clear precipitants that make things worse.’*

(GP17)

Most GPs suggested that beta-blockers were an important *‘tool in the toolbox’* (GP11) for helping patients with generalised anxiety disorder who had physical symptoms. They explained to patients that, although a beta-blocker would not help with their psychological symptoms or *‘cure their anxiety, it can help with the physical symptoms’* (GP8). GPs stated that they explained to patients beta-blockers could also facilitate a positive feedback loop, whereby a reduction in physical symptoms could improve psychological symptoms, if it stopped patients from worrying that physical symptoms might occur:

*‘They stop that kind of cycle of you feeling more stressed and anxious … stop the effects of anxiety that other people can see in you, like shaking and sweating … stops the loop of a vicious circle of kind of feeling more anxious, getting more symptoms, then feeling more anxious.’*

(GP15)

A few GPs said that although they used beta-blockers for situational anxiety, they tended not to prescribe beta-blockers for generalised anxiety disorder. However, they said they would prescribe them if the patient requested beta-blockers, having found them beneficial in the past, or if the patient wanted to try them:

*‘I tend not to unless … the patient is really keen … if they have tried before, then I do in that situation, but I generally do not use them for more generalised anxiety disorders.’*

(GP14)

Most GPs considered psychoeducation or referral to talking therapies as the first-line approach for anxiety. Some GPs said that they would also offer beta-blockers as a first-line drug option, on its own or while the patient was waiting for talking therapies, depending on how severe the anxiety symptoms were:

*‘If their anxiety is quite mild … they are waiting for CBT* [cognitive behavioural therapy] *and particularly if those physical symptoms are particularly bad, I will say, “well this may help you cope with your anxiety attacks” … that’s probably when I would go with them first-line.’*

(GP8)

Many GPs also considered beta-blockers as a second-line treatment *‘where first-line approaches such as SSRIs or CBT have not been effective’* (GP3). Additionally, some talked about using beta-blockers alongside antidepressants as an adjunctive therapy to *‘manage their physical type symptoms’* (GP14):

*‘I have lots of patients who are already on SRRIs, or other antipsychotics, and they actually benefit still from … an adjunct with beta-blockers.’*

(GP10)

#### Beta-blockers are licensed for use in anxiety

Most GPs mentioned beta-blockers were in the *‘BNF licensed for use’* (GP17) for symptoms of anxiety. Around a third of GPs said they knew that beta-blockers were not mentioned in the NICE clinical guidelines for anxiety, with the other GPs saying that they were unsure or *‘did not realise they were not in the NICE guidelines’* (GP2).

GPs were asked why they thought beta-blockers were not in the NICE anxiety guidelines. Some suggested it might be because there was not a *‘very good evidence base for them’* (GP14) or because they only helped with the physical symptoms of anxiety and not the underlying cause:

*‘They do not work very well* [with] *generalised anxiety because they only stop the physical reactions … they do not really change the mental side … perhaps why* [they are] *not in the NICE guideline*[s] *… but perhaps it could be in there as an adjunct to consider when there are lots of physical symptoms.’*

(GP10)

Other GPs were unsure why they were not included and thought they should be included as there was *‘definitely a place for them’* (GP13). Some GPs reflected that perhaps the NICE guidelines were not that pragmatic in terms of medication options available and waiting lists for talking therapies:

*‘I wonder if the guidelines are not always pragmatic and practical, often the guidelines say use CBT, etc., but they are not actually at the coal face …* [patients] *cannot access the psychological interventions. Young people who are concerned* [about] *going on medication quite so young,* [medication] *like SSRIs … we have got to be a bit more pragmatic, and they* [beta-blockers] *work in practice.’*

(GP17)

### Safety-driven prescribing

#### Beta-blockers are ‘low risk’

Many GPs viewed beta-blockers as *‘low-risk’* (GP7) drugs, which were *‘not known to be addictive’* (GP12). Although they noted that they had some side effects, they did not consider them a *‘dangerous drug’* (GP5). Some GPs viewed beta-blockers as a *‘safe alternative’* (GP8) to antidepressants, particularly in young adults where they might be concerned about suicidal ideation:

*‘*[There can be] *Early suicide risk on antidepressants … increasing impulsivity around self-harm, so I guess there is a bigger commitment as a GP to start someone on antidepressants, whereas a beta-blocker feels less risky.’*

(GP16)

When asked about risks or contraindications for prescribing beta-blockers, most GPs talked about the risks for patients with anxiety who had asthma or low blood pressure, who were older, or who were pregnant. However, a few GPs said this did not stop them from prescribing in some of these patient groups, they were just more cautious and would *‘use them in lower doses’* (GP14).

GPs reported a wide of range of doses and durations of prescribing. GPs commented that they were *‘not particularly worried about people being on beta-blockers long term’* (GP11). Indeed, some GPs said they had patients who *‘are on them long term for generalised anxiety disorder … taking them daily’* (GP15). Although there was some sense that they were unsure how much the medication was helping the patient, they said they did not specifically explore this in medication reviews and did not want to stop prescribing *‘something that’s keeping them even, so they tend to stay on it’* (GP12):

*‘Most people that have got it on repeat long term, are actually taking it regularly throughout the day … some people really benefit from that … 2 or 3 years on repeat … so there are patients that are just on it for repeat and you are like, “do you still need to take that … but it’s whether you want to stop it”.’*

(GP1)

Nevertheless, many GPs reported that their patients had some *‘really good results’* (GP10) with beta-blockers and that they could be *‘life-changing’* (GP17).

#### Beta-blockers as an alternative to benzodiazepines

Some GPs viewed beta-blockers as an *‘alternative’* (GP7) to benzodiazepines, particularly if the patient had come into the practice asking for diazepam (a benzodiazepine). They said this was because beta-blockers also acted quickly, like benzodiazepines, but did not lead to dependence:

*‘Our awareness of dependence-forming medicines is growing … there is a big drive to not prescribe* [benzodiazepines] *… in those patients, I might think more about a beta-blocker than a benzo.’*

(GP9)

GPs reflected that there was pressure to not prescribe benzodiazepines and that if a patient did not feel able to wait for talking therapy, and had not responded to, or did not wish to take antidepressants, beta-blockers were often the only other drug available for anxiety. GPs reflected on how difficult it was to access a specialist opinion:

*‘*[prescribing benzodiazepines] *is sort of frowned upon and they* [patients] *can rapidly become dependent … referring to secondary care is not easy, so all the GP’s got left in their armoury really is a beta-blocker.’*

(GP5)

*‘It would be extremely rare for me to give a young person diazepam … that used to be much more common practice historically. So probably* [the increase in beta-blocker prescribing] *is replacing some of that.’*

(GP7)

### Patient-driven prescribing

#### Beta-blockers have an ‘immediate’ effect

Some GPs explained that sometimes patients who consulted for anxiety wanted an immediate improvement in their symptoms and that *‘most people are quite positive about having something to take away’* (GP7). There was a sense that for some patients, it was *‘easier to take a tablet than it is to attend a 40-minute* [therapy] *appointment’* (GP12), even if the tablet would not address the underlying cause of the anxiety.

In addition, some GPs suggested that because of substantial waits for talking therapies, concerns around prescribing benzodiazepines, and *‘2 or 3 weeks for antidepressants to take effect’* (GP5), beta-blockers were the quickest option available to help manage anxiety, particularly if the GP felt under pressure from the patient to prescribe:

*‘The availability of talking therapies is so limited and there is such a long wait … people who are anxious want something now, they do not want something in 9 months … so the pressure to give a drug is really huge. People just feel like they cannot cope … they want somebody to do something now.’*

(GP15)

#### Beta-blockers are not ‘mood-altering’

GPs suggested that some patients seemed to be more willing to try beta-blockers as they were not a *‘mood-altering’* (GP11) drug and potentially *‘more acceptable to patients … they are worried about being dulled’* (GP4). When talking about the other treatment options with patients, GPs explained that they often prescribed beta-blockers for patients who were *‘keen to avoid other forms of treatment, such as antidepressant medication’* (GP14). Some GPs thought that this was because there was still some stigma associated with taking antidepressants, and therefore beta-blockers could be considered less stigmatising. There was also some suggestion that some patients viewed antidepressants as a drug for depression, and not for anxiety:

*‘There is a stigma around taking antidepressants and you explain that antidepressants work for anxiety as well as depression … there is still this massive thing about taking an antidepressant is a sign of failure … whereas if you say … “this is propranolol and it physically stops your heart from racing, therefore may abate your panic attack” … in that group of people that is far more understandable.’*

(GP6)

A few GPs reflected that because they sometimes had concerns about whether patients would take antidepressants for the required length of time for them to work, they might be more likely to prescribe beta-blockers if they thought the patient could benefit:

*‘We start SSRIs and patients just do not really take them …* [which] *sometimes does more harm than good, because a patient will take it for like 4 weeks and then never take it again and then think it does not work and it is like, “well … you need to take it for 6 months* [for it to work]*” … that is probably why we prescribe propranolol more because we know that it does not matter if they just take it for 4 weeks, but actually they might benefit from that.’*

(GP1)

#### Beta-blockers are a ‘patient-managed’ treatment

Most GPs viewed beta-blockers as *‘patient-managed’* (GP13), giving patients control as they could manage the frequency and dose of their medication and were *‘not a long-term commitment, which antidepressants are’* (GP2):

*‘It is much easier to start and stop … sertraline* [is] *harder to stop and start. So, if they wanted to manage their own anxiety medication more it might give them a bit more responsibility … it does help the patient manage their own management plan.’*

(GP13)

GPs said that there could be some patients with situational anxiety who just liked the idea of having a *‘pill in their pocket’*, but they did not know how often these patients actually regularly took the beta-blocker:

*‘People find it really useful to have a pill in their pocket … their bedside drawer … a just-in-case medication … they are really up for that idea.’*

(GP2)

Some GPs explained that because there were no concerns around de-prescribing with beta-blockers in terms of withdrawal effects, there was potentially less need for GP input on managing beta-blocker medication for anxiety, compared with antidepressants or benzodiazepines. They said they were therefore more flexible with the follow-up requirements:

*‘I probably do not have a hard and fast rule* [about follow-up] *… but unlike when prescribing antidepressants when we do have hard and fast rules … I think I am probably a bit looser.’*

(GP17)

Many GPs said that for patients who were being prescribed beta-blockers for situational anxiety, and therefore taking them only in a specific situation to manage their anxiety (for example, for a job interview or exam), they would not necessarily have a follow-up:

*‘If it was a fear of flying … or a presentation fear, then I would say “well, try it first … if it works, well then, I am happy for you to have that on repeat prescription for you to access yourself, come back and see me if there are any problems” … I would be perfectly happy for them to be on it fairly indefinitely.’*

(GP11)

## Discussion

### Summary

GPs view beta-blockers as an important option for helping patients with anxiety to manage physical symptoms. GPs are clear that beta-blockers would not help directly with the psychological symptoms of anxiety or the underlying cause. Many GPs view beta-blockers as a low-risk option compared with antidepressants, particularly in young adults when they might have concerns about the risk of suicidal ideation or increased risk of self-harm behaviours when starting treatment. They are willing to use beta-blockers as a first-line or second-line treatment, or as an adjunct alongside antidepressants. Some GPs also view beta-blockers as an alternative to benzodiazepines, based on the rationale that beta-blockers act quickly, and would not lead to dependence.

GPs suggest that some patients want an immediate improvement in their anxiety symptoms, even if the underlying cause of the anxiety is not being addressed. GPs note there are substantial waits for talking therapies, delays in antidepressants taking effect, and concerns around using benzodiazepines. Therefore, beta-blockers may be seen to be the quickest option available in primary care, while patients wait to receive other treatment. GPs note that some patients seem to be more willing to try beta-blockers than antidepressants, as they are not perceived to be a ‘mental health drug’ that would affect mood, and therefore are potentially more acceptable and less stigmatising for patients. GPs view beta-blockers as a patient-managed treatment, where patients can manage their dose and frequency, with less need for GP input.

### Strengths and limitations

Conducting qualitative interviews allowed participants to raise topics that were salient to them. Participants were sampled purposively and maximum variation was achieved in terms of participants’ age, role, and the practice deprivation decile. Data collection continued until data saturation was reached. However, all interviewees volunteered to be interviewed, and therefore may have been GPs who had particular knowledge of anxiety or were confident in prescribing medication for mental health. Finally, all the GPs interviewed were working in practices in Bristol and the surrounding areas. This was done for convenience purposes, and it is possible that GPs working in other areas of the country might hold different views. That said, we recruited practices from both inner city and rural locations, as well as practices that differed in their deprivation deciles. Finally, although the research team was multidisciplinary, the clinical members of the team did not work directly with the data, coding transcripts. However, they were involved in discussions about the data and in developing the coding frame and naming of themes.

### Comparison with existing literature

Previous research using electronic primary care medical records found that there was a substantial increase in new prescriptions for beta-blockers for patients with anxiety between 2003 and 2018, largely driven by an increase in prescribing in young adults.^7^ The present study suggests that this increase may be caused by several factors, including concerns around prescribing antidepressants in young people and the need for a quick-acting solution. These findings are consistent with previous qualitative research with GPs, which found that media reporting of an association between antidepressants and adolescent suicide potentially influenced GPs’ prescribing habits by making them more cautious about prescribing SSRIs for younger people.^23^ The same study also reported that patients preferred a ‘quick fix’ for their mental health symptoms.^23^ In our study, GPs also suggested that substantial waits to access talking therapy services meant that they prescribed beta-blockers for pragmatic reasons. This finding is in line with other qualitative research with GPs, which reported GPs feeling the pressure to prescribe something in light of long wait lists for talking therapies.^8^ In addition, in our study, GPs reported concerns around benzodiazepine use. Likewise, other studies have described pressures to reduce benzodiazepine prescribing,^24^^–^26 and this is mirrored in quantitative data showing a decline in new benzodiazepine prescriptions in primary care for patients with anxiety.^27^ Further, there is lots of evidence indicating that patients can often feel stigma around taking antidepressants,^28^^,^^29^ which was something GPs in our study were conscious of. Existing research suggests that beta-blockers are widely used for situational anxiety,^30^^,^^31^ and GPs in our study described this as one of the situations where they might prescribe beta-blockers.

A systematic review of the effectiveness of propranolol in treating anxiety disorders concluded that there was inconclusive evidence for the benefit of this drug in treating anxiety, with more high-quality evidence needed.^10^^,^^11^ This may also be why beta-blockers are not mentioned in the NICE clinical guideline for anxiety.^9^ However, in our study, some GPs said they were not aware of this, or were aware but thought that beta-blockers had a role in the management of the physical symptoms of anxiety. This supports findings from other qualitative studies exploring GPs’ views on patients with perinatal anxiety or depression, where GPs described not always following NICE guidance, but using their own experiences to inform treatment decisions.^32^

There have been some recent studies investigating the use of beta-blockers for anxiety disorders,^33^^,^^34^ and beta-blockers have received increased media attention for their use in anxiety.^35^^,^^36^ *The Pharmaceutical Journal* notes that propranolol is reportedly used by celebrities, and states this may be influencing *‘public perceptions and potentially contribute to the normalisation of its use’*.^30^ Conversely, there have also been several reports highlighting concerns around beta-blocker prescribing in people with depression or suicidal ideation, with increasing numbers of propranolol overdoses reported in recent years.^12^^,^^14^ Further, propranolol was added to the watch list of drugs considered to be increasingly available for misuse.^13^ In our study, although GPs talked about the main contraindications for beta-blockers, such as asthma, they did not mention any concerns relating to overdose.

### Implications for research and practice

Although GPs may consider beta-blockers to be safe and useful for the management of anxiety symptoms, there have been recent reports about safety concerns. It is therefore important that GPs are appropriately cautious in their prescribing of beta-blockers for patients who are taking antidepressants or who have a history of suicidal thoughts or attempts. Future research should also seek to understand patients’ views of taking beta-blockers for anxiety. While there are pragmatic reasons for prescribing beta-blockers, they do not feature in the NICE clinical guidelines for anxiety.^9^ It is likely that this is because there is very limited evidence for their effectiveness in treating anxiety.^10^ Given the recent increases seen in primary care prescribing of beta-blockers, there is a clear need for a definitive trial to assess the safety and effectiveness of beta-blockers in treating anxiety. Given the wide range of doses, durations of prescribing, and relevant anxiety presentations described in this study, more than one trial may be required. Evidence from such research would better inform GPs on when, how, and if this drug should be used for the management of people with anxiety.

## Data

Data for this study are not publicly available.
